# Description of Surface Polaritons Within a Classical Coupled Harmonic Oscillator Model

**DOI:** 10.1002/nap2.70238

**Published:** 2026-08-21

**Authors:** Edoardo Vicentini, Nicolas Pajusco, Carlos Maciel‐Escudero, Xabier Arrieta, Javier Aizpurua, Ruben Esteban, Rainer Hillenbrand

**Affiliations:** ^1^ CIC nanoGUNE BRTA Donostia‐San Sebastián Spain; ^2^ Laboratoire d'Acoustique de l'Université du Mans (LAUM) UMR 6613 Institut d'Acoustique—Graduate School (IA‐GS) CNRS Le Mans Université Le Mans France; ^3^ Department of Physics Philipps‐Universität Marburg Marburg Germany; ^4^ Centro de Física de Materiales (CFM‐MPC) CSIC‐EHU Donostia‐San Sebastián Spain; ^5^ Department of Electricity and Electronics University of the Basque Country (EHU) Leioa Spain; ^6^ Donostia International Physics Center (DIPC) Donostia‐San Sebastián Spain; ^7^ IKERBASQUE Basque Foundation for Science Bilbao Spain

**Keywords:** coupled harmonic oscillator model, surface polaritons, ultrastrong coupling

## Abstract

Coupled harmonic oscillator models are widely used to describe strong and ultrastrong light–matter coupling, providing a simple yet powerful framework for the quantitative analysis of hybrid modes across a broad range of physical systems. Surprisingly, this approach has not been applied to describe surface phonon polaritons and surface plasmon polaritons, which are among the most fundamental examples of strongly coupled light–matter modes. Here, we analyze their dispersion relations using a harmonic oscillator model based on momentum coupling. We show that the surface polaritons—hybrid modes derived from classical electromagnetic theory—are exactly reproduced when the uncoupled oscillators are chosen as the surface phonon (or surface plasmon) mode and the dielectric Brewster mode. This identifies the dielectric Brewster mode as the photonic component of surface polaritons—a role that is typically left unspecified in the literature—and demonstrates the relevance of the coupled harmonic oscillator model for describing surface polariton dispersions.

## Graphical abstract

A coupled‐oscillator model describes the formation of surface polaritons. It identifies the dielectric Brewster mode as the photonic constituent of surface polaritons and shows that the familiar surface plasmon or surface phonon polaritons correspond to the lower polariton (LP) branch, whereas Brewster polaritons correspond to the upper polariton (UP) branch. The study provides a complete picture of the surface‐polariton dispersion.

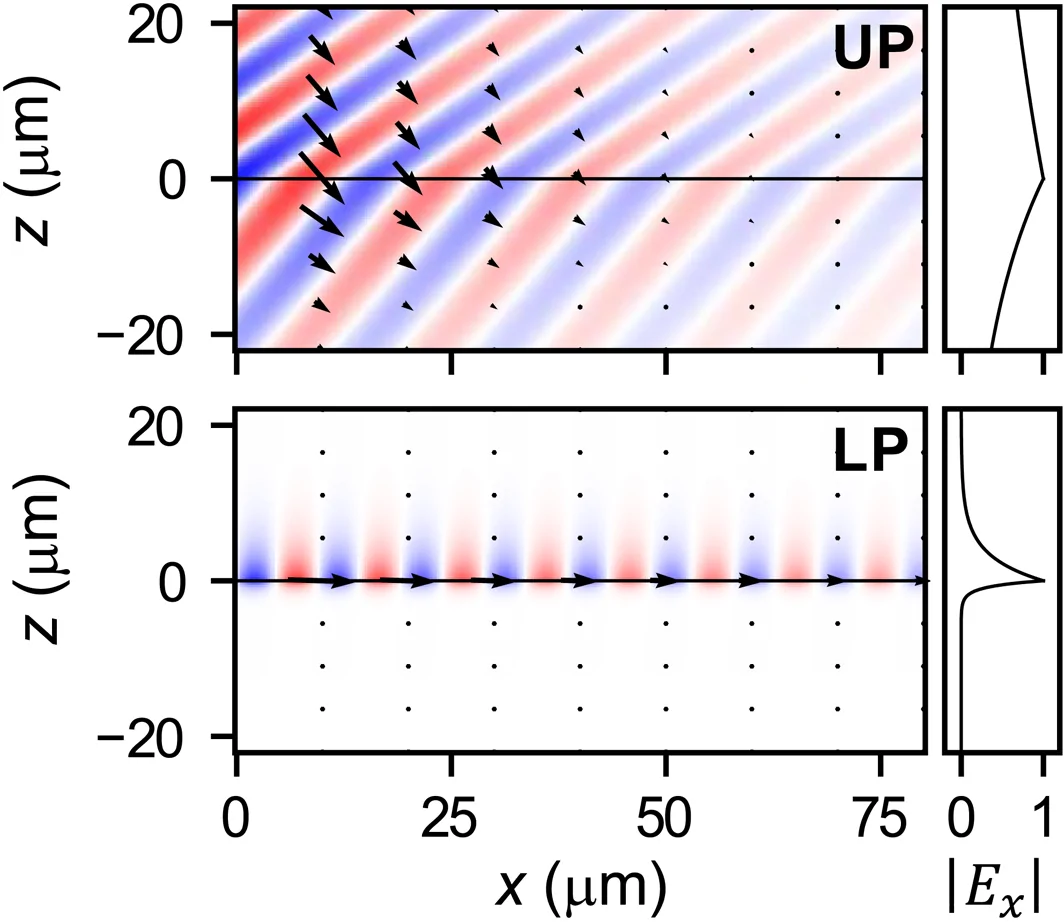

## 1 Introduction

When light interacts coherently with matter—such as an optical cavity mode coupling to an exciton, phonon, or other dipolar material excitation—the system can enter the strong or ultrastrong coupling regime. In this regime, the energy exchange between light and matter is faster than the dissipation rates of the individual components. This coherent interaction produces hybrid modes called polaritons, which are neither purely light nor purely matter. These mixed light–matter states exhibit properties that differ fundamentally from those of their uncoupled constituents and enable a wide range of phenomena in photonics, condensed matter physics, and quantum optics [1, 2, 3, 4, 5, 6].

A simple way to describe these hybrid modes is through classical harmonic oscillator models (CHOMs) [7, 8, 9]. In this framework, the optical mode and the material excitation are each treated as independent oscillators, and their coupling gives rise to hybridized modes that correspond to the upper and lower polariton branches. Analogous to the quantum description of light–matter interaction provided by the Hopfield Hamiltonian [10], the coupling between the optical and material modes is characterized by the coupling strength *g*. Distinct coupling regimes are defined by comparing g with the losses and resonance frequencies of the uncoupled oscillators. Strong coupling (SC) occurs when g exceeds the damping rates of the individual modes, whereas the ultrastrong coupling (USC) regime is reached when g becomes a significant fraction of the mode frequencies themselves [1, 2, 3, 4, 5, 6].

Several variants of the CHOM exist [11], with the choice depending on the coupling regime and on whether the (diamagnetic) term, present in the Hopfield Hamiltonian and originating from the minimal‐coupling light–matter Hamiltonian, is significant. The most commonly used formulations are the spring‐coupling model and the momentum‐coupling (MoC) model, the latter accurately describing strong and ultrastrong coupling between light and transverse optical phonons in polar crystals, leading to the formation of volume phonon polaritons [11, 12, 13]. The classical harmonic oscillator models provide results fully consistent with the quantum description in regimes where the system does not require a quantum‐mechanical treatment and quantum effects can be neglected.

Despite their simplicity, CHOMs have been remarkably successful in describing SC and USC in various systems, ranging from molecules in optical cavities to exciton–polariton modes in semiconductors [2, 3, 14, 15]. Surprisingly, they have not yet been applied to analyze the canonical surface polariton.

Surface polaritons are electromagnetic (EM) waves resulting from the coupling of light with collective matter excitations at the surface of a medium [16, 17, 18, 19, 20, 21]. Common examples include surface plasmon polaritons (SPPs) [22, 23, 24], which involve collective oscillations of free carriers in metals or semiconductors, and surface phonon polaritons (SPhPs) [25, 26, 27], which arise from lattice vibrations in polar crystals. These surface polaritons can be localized or propagating along the interface, with strong field confinement perpendicular to the interface and strongly enhanced fields near the interface. Their wavelengths are shorter than that of free‐space photons at the same frequency, enabling subwavelength control of light and making them highly useful for sensing, nanophotonic devices, and light–matter interaction at the nanoscale [26, 28, 29, 30, 31, 32, 33].

Here, we apply the MoC model to revisit SPhPs and SPPs. We address key questions that are rarely discussed in the literature, including the identification of the bare (uncoupled) light mode that hybridizes to form surface polaritons, the complementary polariton branch, and the coupling strength. Starting from dispersions derived from the solutions of Maxwell's equations, we show that SPhPs and SPPs can be understood as the result of strong coupling between the surface phonon or plasmon and the dielectric Brewster mode, the bare EM wave propagating along the surface at the Brewster condition in the absence of phonons or free carriers. Within the MoC model, the conventional surface polariton corresponds to the lower polariton branch, whereas its upper branch, which we term the Brewster polariton (BrP), is a weakly confined surface mode. This analysis offers insight into surface polariton formation and reveals the connection between strongly confined surface polaritons and weakly confined Brewster polaritons arising from strong light–matter coupling.

## 2 Results

We first briefly review the standard electromagnetic description of surface polaritons dispersion [16, 17, 18, 19, 20, 21, 34]. Surface polaritons correspond to transverse magnetic (TM) surface wave solutions of Maxwell's equations that exist at the interface between two distinct media (Figure 1a). For a TM mode at the boundary, z=0, between two isotropic nonmagnetic media, the in‐plane dispersion relation is given by (additional details about the derivation are provided in Supporting Information S1: Note 1 and Note 2):

(1) $$k_{x}=\frac{\omega }{c_{0}}\sqrt{\;\frac{\varepsilon_{1}\varepsilon_{2}}{\varepsilon_{1}+\varepsilon_{2}}},$$

where $k_{x}$ is the in‐plane wavevector component, $\varepsilon_{1}$ and $\varepsilon_{2}$ are the dielectric functions of the two media forming the interface, ω is the angular frequency and $c_{0}$ is the speed of light in vacuum. From the in‐plane wavevector, the out‐of‐plane wavevector component in each medium is obtained as

(2) $$k_{z,j}=\sqrt{\varepsilon_{j}k_{0}^{2}-k_{x}^{2}},$$

where $k_{0}=\omega /c_{0}$ is the magnitude of the free‐space wavevector and j=1,2 denotes the respective medium.

**FIGURE 1:**
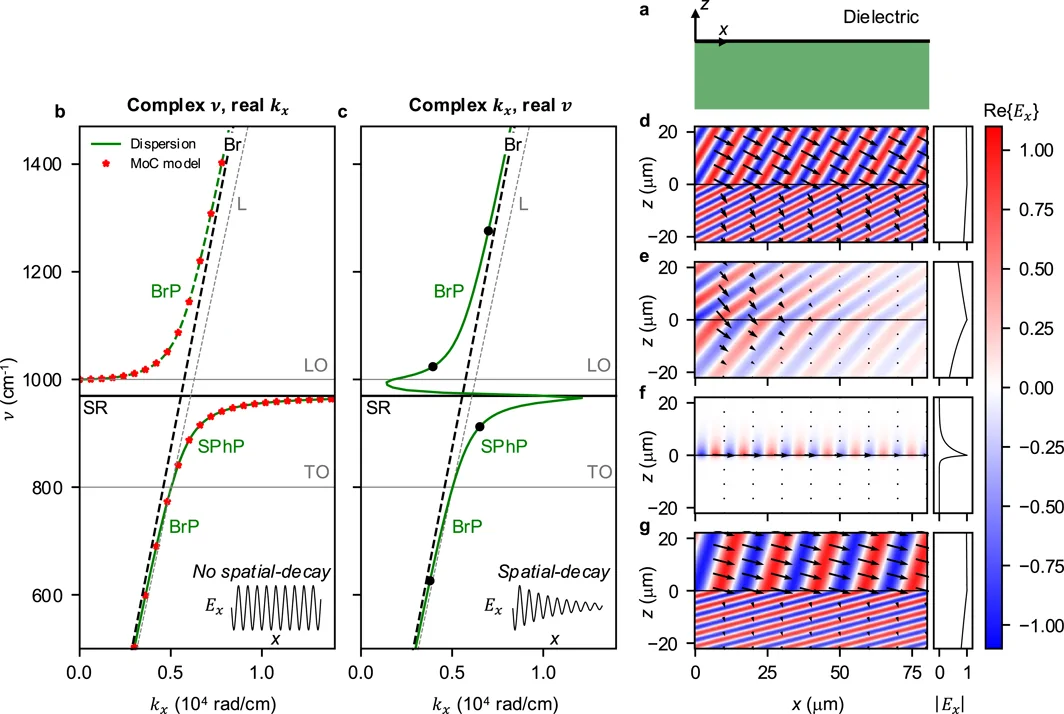
Surface phonon polariton dispersion. (a) Illustration of the boundary between a dielectric and a phononic material which supports SPhPs. (b) Surface mode dispersion calculated for real wavevector and complex frequency. The lower polariton branch is shown as a solid green line and the upper polariton branch as a dashed green line, obtained both from the Maxwell dispersion relation (Equation 9). Red stars mark the mode frequencies of the MoC model (solution of Equation 8). (c) Surface mode dispersion calculated for real frequencies and complex wavevectors. Black dots indicate the frequencies and in‐plane wavevectors for which panels (d–g) are calculated. (d, e, f, g) Real part of the normalized electric field $E_{x}\left(x,z\right)$ as function of x and z at (d) ν = 1275 cm^−1^, (e) ν = 1025 cm^−1^, (f) ν = 912 cm^−1^, and (g) ν = 626 cm^−1^. Black arrows indicate the Poynting vector. Right panels show the corresponding spatial decay of ${\left|E_{x}\right|}^{2}$ along the z‐axis. (b–g) Dispersion curves and field profiles are calculated using Equations (1) and (3) with parameters: $\varepsilon_{1}$ = 1, $\varepsilon_{\infty }$ = 5, $\nu_{\text{TO}}$ = 800 cm^−1^, $\nu_{\text{LO}}$ = 1000 cm^−1^, and $\gamma_{\text{TO}}$ = 10 cm^−1^. (d–g) Fields plots are normalized to their maximum/minimum as ± 1. Br: dielectric Brewster mode (dashed black line); BrP: Brewster polariton; L: light line in vacuum (dashed gray line); LO: longitudinal optical phonon (solid gray line); SPhP: surface phonon polariton; SR: surface resonance (solid black line); and TO: transverse optical phonon (solid gray line).

### 2.1 Surface Phonon Polaritons

SPhPs are a class of surface polaritons that arise at the interface between a dielectric (usually vacuum) and a polar material supporting optical phonons (Figure 1a). The dielectric response of the phononic medium can be described using a Lorentz oscillator model [17, 18, 19, 20, 21]:

(3) $$\varepsilon_{2}=\varepsilon_{\text{Ph}}\left(\omega \right)=\varepsilon_{\infty }+\left(\frac{S}{\omega_{\text{TO}}^{2}{-\omega }^{2}-i\gamma_{\text{TO}}\omega }\right)=\varepsilon_{\infty }\left(\frac{\omega^{2}-\omega_{\text{LO}}^{2}+i\gamma_{\text{TO}}\omega }{\omega^{2}-\omega_{\text{TO}}^{2}+i\gamma_{\text{TO}}\omega }\right),$$

where $\varepsilon_{\infty }$ is the background permittivity, $\omega_{\text{TO}}$ and $\omega_{\text{LO}}=\sqrt{\omega_{\text{TO}}^{2}+S/\varepsilon_{\infty }}$ are the transverse and longitudinal optical phonon frequencies, respectively, *S* is the oscillator strength, and $\gamma_{\text{TO}}$ is the phonon damping rate.

The dispersion relation in Equation (1) can be solved [21, 35, 36] either by imposing real in‐plane wavevectors, $k_{x}$, yielding complex frequencies, $\tilde{\omega }=\omega -i\gamma /2$, or, conversely, by imposing real frequencies, ω, yielding complex in‐plane wavevectors, ${\tilde{k}}_{x}=k_{x}+i\kappa$. Both descriptions encode the same physics—loss, confinement, and propagation—but emphasize different aspects of the electromagnetic wave. Solving for real $k_{x}$ yields complex eigenfrequencies, whose real part, ω, gives the oscillation frequency whereas the imaginary part, γ, describes the temporal decay rate. This formulation is commonly used to analyze resonances and quasi‐normal modes. In contrast, solving for real ω is more convenient when studying spatial propagation and attenuation lengths.

Figure 1b,c present the surface modes dispersions obtained from Equation (1) for an interface between a dielectric with $\varepsilon_{1}=1$ and a phononic medium (with material parameters similar to 3C‐SiC) described by Equation (3) with $\varepsilon_{\infty }=5$, $\nu_{\text{TO}}=$ 800 cm^−1^, $\nu_{\text{LO}}=$ 1000 cm^−1^, and $\gamma_{\text{TO}}=10$ cm^−1^ (with ν=ω/2πc). When the dispersion is computed for real wavevectors with complex frequencies (Figure 1b), two distinct dispersion branches emerge. One branch (green solid line) lies below the surface phonon resonance (SR) frequency, $\omega_{\text{SR}}$, (given by $\varepsilon_{\text{Ph}}\left(\omega_{\mathrm{SR}}\right)=-\varepsilon_{1}$), and the other (green dashed line) lies above the longitudinal optical phonon frequency $\omega_{\text{LO}}$. These dispersion branches are separated by a gap between $\omega_{\text{SR}}$ and $\omega_{\text{LO}}$. In contrast, solving for real frequencies and complex wavevectors (Figure 1c), produces a single continuous dispersion curve, which exhibits a characteristic back‐bending between $\omega_{\text{SR}}$ and $\omega_{\text{LO}}$ [16].

Typically, only the portion of the dispersion lying within the Reststrahlen band, where $\text{Re}\left[\varepsilon_{\text{Ph}}\right]<0$, is of interest. In this frequency range between $\omega_{\text{TO}}$ and $\omega_{\text{SR}},$ the mode is referred to as a SPhP, characterized by in‐plane wavevector exceeding that of free‐space light, strong field confinement perpendicular to the surface, and large field enhancement. The other parts of the dispersion—below $\omega_{\text{TO}}$ and above $\omega_{\text{LO}}$—are often ignored or described inconsistently in the literature [16, 17, 18, 19, 20, 21, 36]. In particular, the branch above $\omega_{\text{LO}}$ (dashed green line) is sometimes referred to as a Brewster mode or omitted. Rather than being disregarded, these neglected branches warrant careful consideration as viewing them together with the SPhP branch clearly resembles a typical light–matter coupling scenario. The upper (dashed green) and lower (solid green) branches exhibit the characteristic anticrossing of strong coupling, along with a polaritonic gap. At large in‐plane wavevectors $k_{x}$, the branches asymptotically approach the bare modes. The lower branch tends toward the surface phonon resonance (SR), defined in a lossless phononic material as

(4) $$\omega_{\text{SR}}=\sqrt{\frac{\varepsilon_{\infty }\;\omega_{\text{LO}}^{2}+\varepsilon_{1}\;\omega_{\text{TO}}^{2}}{\varepsilon_{1}+\varepsilon_{\infty }}},$$

and shown as the horizontal black line in Figure 1b, whereas the upper branch approaches a mode with linear dispersion (dashed black line),

(5) $$\omega_{\text{Br}}\left(k_{x}\right)=c_{0}\;k_{x}\;\sqrt{\frac{\varepsilon_{1}+\varepsilon_{\infty }}{\varepsilon_{1}\;\varepsilon_{\infty }}},$$

corresponding to a TM‐polarized light wave satisfying the Brewster condition (zero surface reflection) in the absence of phonons. We denote this mode as the dielectric Brewster mode (Br), $\omega_{\text{Br}}\left(k_{x}\right)$. The upper polariton branch (dashed green line in Figure 1b) is consequently referred to as the upper Brewster polariton (BrP) with dispersion $\omega_{\text{BrP}}\left(k_{x}\right)$. The lower polariton branch consists of two segments: between $\omega_{\text{TO}}$ and $\omega_{\text{SR}}$, it corresponds to the well‐known surface phonon polariton (SPhP) with dispersion $\omega_{\text{SPhP}}\left(k_{x}\right)$, whereas below $\omega_{\text{TO}}$, it again exhibits a Brewster‐like character. We thus refer to this segment below $\omega_{\text{TO}}$ as the lower Brewster polariton.

To demonstrate that the entire surface‐mode dispersion can be accurately modeled by SC between the surface phonon resonance and the dielectric Brewster mode, we apply a well‐established coupled harmonic oscillator model (CHOM). In the later Discussion section, we analyze the electromagnetic properties of the modes corresponding to the upper and lower dispersion branches—including the field distribution and Poynting vector—to verify that interpreting them as Brewster polaritons is physically meaningful and fully consistent with Maxwell's equations.

We employ the momentum coupling (MoC) model, which can describe both SC and USC between a light mode and a matter (dipolar) excitation [11]. The bare matter excitation is described by a general coordinate $x_{2}$ and eigenfrequency $\omega_{2}$, and includes a loss term $\gamma_{2}$. The light mode is described by a general coordinate $x_{1}$ and eigenfrequency $\omega_{1}$ and is considered lossless. The corresponding coupled differential equations are given by [11]:

(6) $${\ddot{x}}_{1}+\omega_{1}^{2}x_{1}+2g{\dot{x}}_{2}=0,$$

(7) $${\ddot{x}}_{2}+\omega_{2}^{2}x_{2}+\gamma_{2}{\dot{x}}_{2}-2g{\dot{x}}_{1}=0$$

where g is the coupling constant that quantifies the strength of the interaction between the two bare oscillators, and the dot denotes the time derivative. Solving this system of equations in frequency domain yields the hybrid mode frequencies $\omega_{\pm }$ from the characteristic equation

(8) $$\omega^{4}+i\gamma_{2}\omega^{3}-\left(\omega_{1}^{2}+\omega_{2}^{2}+{4g}^{2}\right)\omega^{2}-i\gamma_{2}\omega_{1}^{2}\omega +\omega_{1}^{2}\omega_{2}^{2}=0.$$

The hybrid mode frequencies $\omega_{\pm }$ correspond to the roots of Equation (8). The different coupling regimes are defined based of the coupling constant g. When $g>\gamma_{2}/4$, the system is in the SC regime and when $g>0.1\;\omega_{2}$, the system is in the USC regime [11].

To compare Equation (8) with the dispersion relation obtained from Maxwell's equations, we calculate the surface mode starting from Equation (1) by substituting the dielectric function of the polar material, $\varepsilon_{2}=\varepsilon_{\text{Ph}}$ (Equation 3). By further inserting the expressions for $\omega_{\text{SR}}$ from Equation (4) and $\omega_{\text{Br}}$ from Equation (5), we derive the characteristic equation governing the surface‐mode dispersion:

(9) $$\omega^{4}+i\gamma \omega^{3}-\left(\omega_{\text{Br}}^{2}+\omega_{\text{SR}}^{2}+\left(\omega_{\text{LO}}^{2}-\omega_{\text{SR}}^{2}\right)\right)\omega^{2}-i\gamma \omega_{\text{Br}}^{2}\omega +\omega_{\text{Br}}^{2}\omega_{\text{SR}}^{2}=0,$$

whose roots yield the lower and upper dispersion branches as shown in Figure 1b.

Comparing the MoC model, Equation (8), and Maxwell's characteristic equation, Equation (9), we find that the hybrid mode frequencies $\omega_{\pm }$ of the MoC model correspond exactly to $\omega_{\text{SPhP}}$ and $\omega_{\text{BrP}}$ when:

(10) $$\omega_{1}=\omega_{\text{Br}},$$

(11) $$\omega_{2}=\omega_{\text{SR}},$$

(12) $$g=\sqrt{\frac{\omega_{\text{LO}}^{2}-\omega_{\text{SR}}^{2}}{4}}=\sqrt{\frac{\left(\omega_{\text{LO}}^{2}-\omega_{\text{TO}}^{2}\right)}{4}\left(\frac{\varepsilon_{1}}{{\varepsilon_{\infty }+\varepsilon }_{1}}\right)}.$$

This analysis shows that the surface phonon polariton (SPhP) and the Brewster polariton can be described as arising from the coupling between the surface phonon resonance (SR) and the dielectric Brewster mode (Br). Substituting these parameters into the MoC dispersion relation, Equation (8), yields the red stars in Figure 1b, which show perfect agreement with the electromagnetic solution and thereby validate the coupled harmonic oscillator picture.

### 2.2 Surface Plasmon Polaritons

Before entering the discussion of the physical nature of the polariton modes, we repeat the same analysis done for SPhP for the surface plasmon polaritons, showing that the results obtained from the coupled harmonic oscillator model are general and not a unique property of the phonons.

SPPs are surface polaritons that form at a dielectric–metal interface. By modeling the metal with a Drude dielectric function [17, 18, 19, 20, 21], the permittivity is expressed by:

(13) $$\varepsilon_{2}=\varepsilon_{m}\left(\omega \right)=\varepsilon_{\infty }\left(1-\frac{\omega_{P}^{2}}{\omega^{2}+i\gamma_{P}\omega }\right),$$

where $\varepsilon_{\infty }$ is the background permittivity and

(14) $$\omega_{P}^{2}=\frac{ne^{2}}{m_{\text{eff}}\varepsilon_{0}\varepsilon_{\infty }}$$

is the screened plasma frequency of the metal with n the free carrier density, $m_{\text{eff}}$ the carrier effective mass, e the electron charge, $\varepsilon_{0}$ the vacuum permittivity, and $\gamma_{P}$ the plasma damping.

Figure 2b,c show the mode dispersions obtained from Equation (1) for an interface between a dielectric with $\varepsilon_{1}=1$ and a Drude metal defined by Equation (13) with $\varepsilon_{\infty }=5$, $\nu_{P}$ = 1000 cm^−1^, and $\gamma_{P}=20$ cm^−1^. Similarly to SPhPs, when the dispersion is computed as real in‐plane wavevector with complex frequencies (Figure 2b), two distinct dispersion branches emerge, one for $\omega <\omega_{\text{SR}}$ (blue solid line), where the surface plasmon resonance (SR) corresponds to the surface plasma frequency of a lossless metal

(15) $$\omega_{\text{SR}}=\omega_{P}\sqrt{\frac{\varepsilon_{\infty }}{\varepsilon_{1}+\varepsilon_{\infty }}},$$

and one for $\omega >\omega_{P}$ (blue dashed line). These dispersion branches are separated by a gap between $\omega_{\text{SR}}$ and $\omega_{P}$. In contrast, solving for complex in‐plane wavevector at real frequencies (Figure 2c) produces a single continuous branch exhibiting a characteristic back‐bending between $\omega_{\text{SR}}$ and $\omega_{P}$. Also, in this case, only the part of the dispersion corresponding to $\text{Re}\left[\varepsilon_{m}\right]<0$ ($\omega <\omega_{P}$ ) is considered as the surface plasmon polariton, whereas the upper branch is ignored or described inconsistently [16, 17, 18, 19, 20, 21, 36].

**FIGURE 2:**
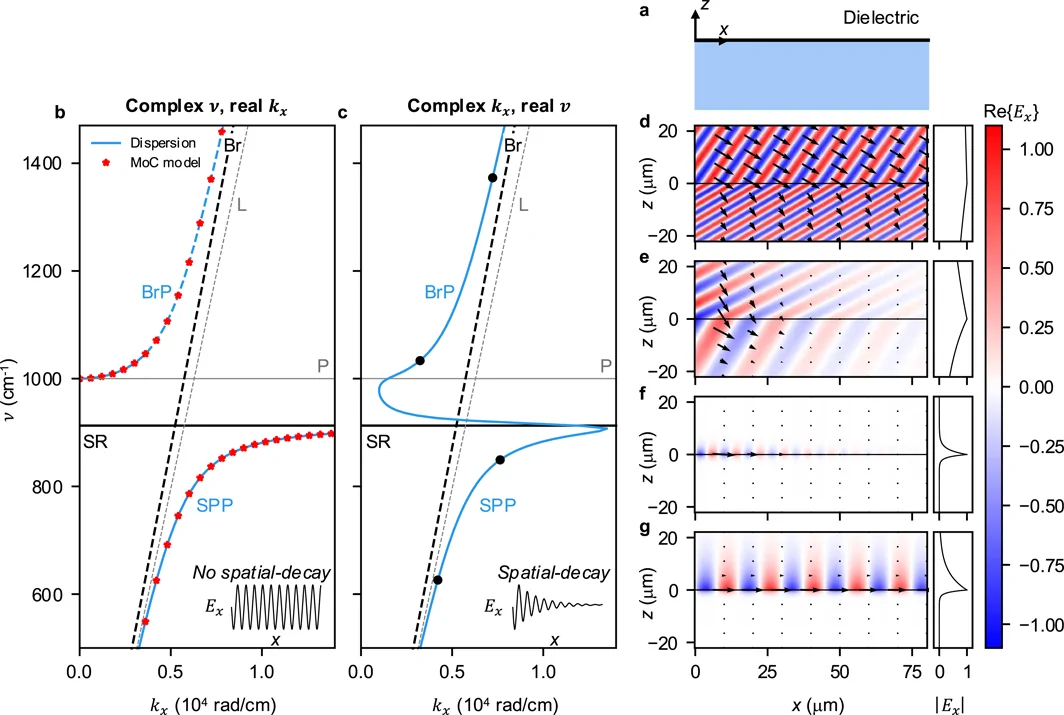
Surface plasmon polariton dispersion. (a) Illustration of the boundary between a dielectric and a metal which supports SPPs. (b) Surface mode dispersion calculated for real wavevector and complex frequency. The lower polariton branch is shown as a solid blue line and the upper polariton branch as a dashed blue line, both obtained from the Maxwell dispersion relation (Equation 16). Red stars mark the hybrid mode frequencies of the MoC model (solution of Equation 8). (c) Surface mode dispersion calculated for real frequencies and complex wavevectors. Black dots indicate the frequencies and in‐plane wavevectors for which panels (d–g) are calculated. (d, e, f, g) Real part of the normalized electric field $E_{x}\left(x,z\right)$ as function of x and z at (d) ν = 1373 cm^−1^, (e) ν = 1033 cm^−1^, (f) ν = 849 cm^−1^, and (g) ν = 626 cm^−1^. Black arrows indicate the Poynting vector. Right panels show the corresponding spatial decay of ${\left|E_{x}\right|}^{2}$ along the z‐axis. (b, c) Dispersion curves and field profiles are calculated using Equations (1) and (13) with parameters: $\varepsilon_{1}$ = 1, $\varepsilon_{\infty }$ = 5, $\nu_{P}$ = 1000 cm^−1^, and $\gamma_{P}=20$ cm^−1^. (d–g) Fields plots are normalized to their maximum/minimum as ± 1. Br: dielectric Brewster mode (dashed black line); BrP: Brewster polariton; L: light line in vacuum (dashed gray line); P: screened plasmon (solid gray line); and SPP: surface plasmon polariton; SR: Surface plasmon (solid black line).

Similarly to the SPhP case, the surface plasmon dispersion curves in Figure 2b clearly resemble a typical strong light–matter coupling scenario. The upper (dashed blue) and lower (solid blue) branches exhibit the characteristic anticrossing of strong coupling, along with a polaritonic gap. At large in‐plane wavevectors $k_{x}$, the branches asymptotically approach the bare modes. The lower branch correspons to the well‐known surface plasmon polariton (SPP) with dispersion $\omega_{\text{SPP}}$ and tends toward the surface plasmon $\omega_{\text{SR}}$. The upper branch, which we denote as Brewster polariton (BrP) with dispersion $\omega_{\text{BrP}}$, approaches a mode with linear dispersion (dashed black line), $\omega_{\text{Br}}\left(k_{x}\right)$ (Equation 5), the dielectric Brewster mode.

Analogously to the SPhP analysis, we obtain the characteristic equation for the surface‐mode dispersion by inserting Equation (13) into Equation (1), leading to

(16) $$\omega^{4}+i\gamma_{P}\omega^{3}-\left(\omega_{\text{Br}}^{2}+\omega_{\text{SR}}^{2}+\left(\omega_{P}^{2}-\omega_{\text{SR}}^{2}\right)\right)\omega^{2}-i\gamma_{P}\omega_{\text{Br}}^{2}\omega +\omega_{\text{Br}}^{2}\omega_{\text{SR}}^{2}=0,$$

whose roots with positive real part yield the dispersions $\omega_{\text{SPP}}\left(k_{x}\right)$ and $\omega_{\text{BrP}}\left(k_{x}\right)$ in Figure 2b. Comparing Equations (8) and (16), we find that the hybrid modes frequencies $\omega_{\pm }$ of the MoC model correspond exactly to $\omega_{\text{BrP}}$ and $\omega_{\text{SPP}}$ when:

(17) $$\omega_{1}=\omega_{\text{Br}},$$

(18) $$\omega_{2}=\omega_{\text{SR}},$$

(19) $$g=\sqrt{\frac{\omega_{P}^{2}-\omega_{\text{SP}}^{2}}{4}}=\sqrt{\frac{\omega_{P}^{2}}{4}\left(\frac{\varepsilon_{1}}{{\varepsilon_{\infty }+\varepsilon }_{1}}\right)}.$$

This analysis shows that the surface plasmon polariton (SPP) and the Brewster polariton (BrP) can be described as arising from the coupling between the surface plasmon resonance (SR) and the dielectric Brewster mode (Br). Substituting these parameters into the MoC dispersion relation, Equation (8) yields the red stars in Figure 2b, which show perfect agreement with the electromagnetic solution.

## 3 Discussion

Within the CHOM, the coupling between the surface phonon/plasmon resonance (SR) and the dielectric Brewster mode (Br) gives rise to two hybrid polariton branches: the upper polariton, which we coin as the (upper) Brewster polariton, and the lower polariton, corresponding partially (above TO frequency in case of phonons) or entirely (in case of plasmons) to the well‐known surface phonon/plasmon polariton (SPhP/SPP). On the other hand, within electromagnetic theory, surface polaritons, such as SPhPs and SPPs, are usually identified as poles of the reflection coefficient, whereas the Brewster polariton corresponds to a zero of the reflection coefficient for p‐polarized light and thus has been often not considered a surface polariton. Indeed, compared with SPhPs and SPPs, which are strongly confined, the Brewster polariton is weakly confined at the interface and has therefore received less attention in surface‐polariton physics and nanophotonics. However, rigorous mathematical analyses suggest that, when evaluated on the appropriate Riemann sheet, it also corresponds to a pole of the reflection coefficient [18, 34, 37, 38]. As such, the Brewster polariton qualifies as a surface polariton. This fact supports the outcome of the CHOM analysis, specifically, that the Brewster polariton is the upper counterpart to SPhPs and SPPs.

To further evaluate the surface‐mode character of the Brewster polariton, we show its spatial field distribution, $\text{Re}\{E_{x}\left(x,z\right)\}$, and the time‐averaged Poynting vector in Figures 1d,e,g and 2d,e. The Brewster polariton propagates along the interface and its field decays exponentially in the out‐of‐plane direction. In contrast to the tightly bound SPhP and SPP modes (Figures 1f and 2f,g), it decays over many wavelengths and exhibits oscillatory behavior into both media, indicating significantly weaker confinement. The Poynting vector crosses the surface but vanishes far above and below, confirming that vertical energy transfer is confined to the interface. Thus, the Brewster polariton is a weakly confined surface mode, which arises from strong light–matter coupling in presence of a surface.

To establish the physical meaning of the CHOM bare modes, we relate them to specific light and matter excitations. The bare mode with linear dispersion, forming the asymptote of the upper (Brewster) polariton branch, corresponds to the dielectric Brewster mode. This mode describes an electromagnetic wave propagating along the interface in the absense of the material resonance (phonons or free carriers) and therefore represents the bare light mode. Conversely, the bare mode with horizontal dispersion corresponds to the surface phonon or surface plasmon resonance, which represents a bare matter mode (Supporting Information S1: Note 3 shows the calculation of this surface resonance from Maxwell's equations in the electrostatic limit). Together, these correspondences demonstrate that the CHOM bare modes are not merely formal constructs but reflect meaningful bare light and matter excitations.

To verify the expression for $g_{\text{SPhP}}$ and $g_{\text{SPP}}$ (Equations 12 and 19) obtained from the comparison of the MoC model to the solution of Maxwell's equations, we derive them alternatively from the ratio of the electric field energy of the bare light mode (dielectric Brewster mode) within the region of space where matter excitations are present (lower half space) to the total electromagnetic energy of the bare light mode in the whole space [11, 12, 35, 39], according to

(20) $$g_{\text{surface}}=g_{\text{bulk}}\sqrt{\frac{2W_{l}}{U_{\text{total}}}},$$

where $g_{\text{bulk}}$ is the light matter coupling strength in bulk,

(21) $$W_{l}=\int_{l}\frac{\varepsilon_{0}\varepsilon_{\infty }}{2}{\left|E_{l}\right|}^{2}dV,$$

is the electric energy in the lower half‐space with $E_{l}=\left(E_{l,x},0,E_{l,z}\right)$ the electric field and

(22) $$U_{\text{total}}=\int_{l}\left[\frac{\varepsilon_{0}\varepsilon_{\infty }}{2}{\left|E_{l}\right|}^{2}+\frac{\mu_{0}}{2}{\left|H_{l}\right|}^{2}\right]dV+\int_{u}\left[\frac{\varepsilon_{0}\varepsilon_{1}}{2}{\left|E_{u}\right|}^{2}+\frac{\mu_{0}}{2}{\left|H_{u}\right|}^{2}\right]dV,$$

the total electromagnetic energy in both half‐spaces, with the upper half space described by the magnetic field $H_{u}=\left(0,H_{u,y},0\right)$, and the electric field $E_{u}=\left(E_{u,x},0,E_{u,z}\right)$, $\varepsilon_{0}$ the vacuum permittivity, $\mu_{0}$ the vacuum permeability, and dV indicated the integral over the volume. Since the dielectric Brewster mode is not decaying along the *z*‐direction, the integral in Equations (21) and (22) over a semi‐infinite domain is diverging. To avoid the divergence, we compute the dielectric Brewster mode with a small finite loss in the lower medium and take the limit of vanishing loss. This yields

(23) $$\frac{2W_{l}}{U_{\text{total}}}=\frac{\frac{1}{k_{z,2}}}{\frac{1}{k_{z,1}}+\frac{1}{k_{z,2}}}=\frac{\varepsilon_{1}}{\varepsilon_{1}+\varepsilon_{\infty }},$$

such that we obtain

(24) $$g_{\text{surface}}=g_{\text{bulk}}\sqrt{\frac{\varepsilon_{1}}{{\varepsilon_{1}+\varepsilon }_{\infty }}}.$$

Equation (24) highlights that surface polaritons have a reduced coupling strength compared to bulk polaritons, since matter and light overlap in only one half of the full space.

For phonons [11, 12, 35], $g_{\text{bulk}}=\frac{1}{2}\sqrt{\omega_{\text{LO}}^{2}-\omega_{\text{TO}}^{2}}$, and expressing $\omega_{\text{TO}}$ via the surface phonon frequency $\omega_{\text{SR}}$ and using equation 4 finally yields

(25) $$g_{\text{SPhP}}=\frac{1}{2}\sqrt{\omega_{\text{LO}}^{2}-\omega_{\text{TO}}^{2}}\sqrt{\frac{\varepsilon_{1}}{{\varepsilon_{1}+\varepsilon }_{\infty }}}=\frac{1}{2}\sqrt{\omega_{\text{LO}}^{2}-\omega_{\text{SR}}^{2}}.$$

For plasmons [40], $g_{\text{bulk}}=\frac{1}{2}\omega_{P}$, yielding

(26) $$g_{\text{SPP}}=\frac{1}{2}\omega_{P}\;\sqrt{\frac{\varepsilon_{1}}{{\varepsilon_{1}+\varepsilon }_{\infty }}}=\frac{1}{2}\sqrt{\omega_{P}^{2}-\omega_{\text{SR}}^{2}},$$

Both Equations (25) and (26) are identical to the coupling strengths obtained from the comparison of the MoC model to the solution of Maxwell's equations (Equations 12 and 19, respectively).

We finally analyze the conditions required for the formation of SPPs and SPhPs. Figure 3 presents phase diagrams of light–matter coupling regimes for volume (VPs) and surface polaritons (SPs). The vertical and horizontal axes correspond to the normalized damping $\gamma /\sqrt{\omega_{\text{LO}}^{2}-\omega_{\text{TO}}^{2}}$ and the high‐frequency permittivity $\varepsilon_{\infty }$, respectively. The three columns correspond to different oscillator strengths characterized by the ratio $\omega_{\text{TO}}/\sqrt{\omega_{\text{LO}}^{2}-\omega_{\text{TO}}^{2}}$. The left column ($\omega_{\text{TO}}=0$) represents metals. In this case, the Lorentz dielectric function of Equation (3) reduces to the Drude dielectric function, such that $\omega_{\text{LO}}$ becomes the screened plasma frequency. The central column represents strong oscillators, such as optical phonons in polar crystals, where $\omega_{\mathrm{TO}}^{2}∼\omega_{\mathrm{LO}}^{2}-\omega_{\mathrm{TO}}^{2}$ are comparable. The right column represents weak oscillators, such as molecular vibrations, for which $\omega_{\mathrm{TO}}^{2}\gg \omega_{\mathrm{LO}}^{2}-\omega_{\mathrm{TO}}^{2}$. The upper row (Figure 3a–c) corresponds to bulk polaritons, whereas the lower row (Figure 3d–f) corresponds to surface polaritons. The phase diagrams summarize the regions where the conditions for strong coupling (SC), ultrastrong coupling (USC), Reststrahlen‐band (RB) formation, volume polaritons (VPs), surface resonances (SRs), and surface polaritons (SPs) are satisfied. Supporting Information S1: Note 4 describes how the phase diagram is calculated.

**FIGURE 3:**
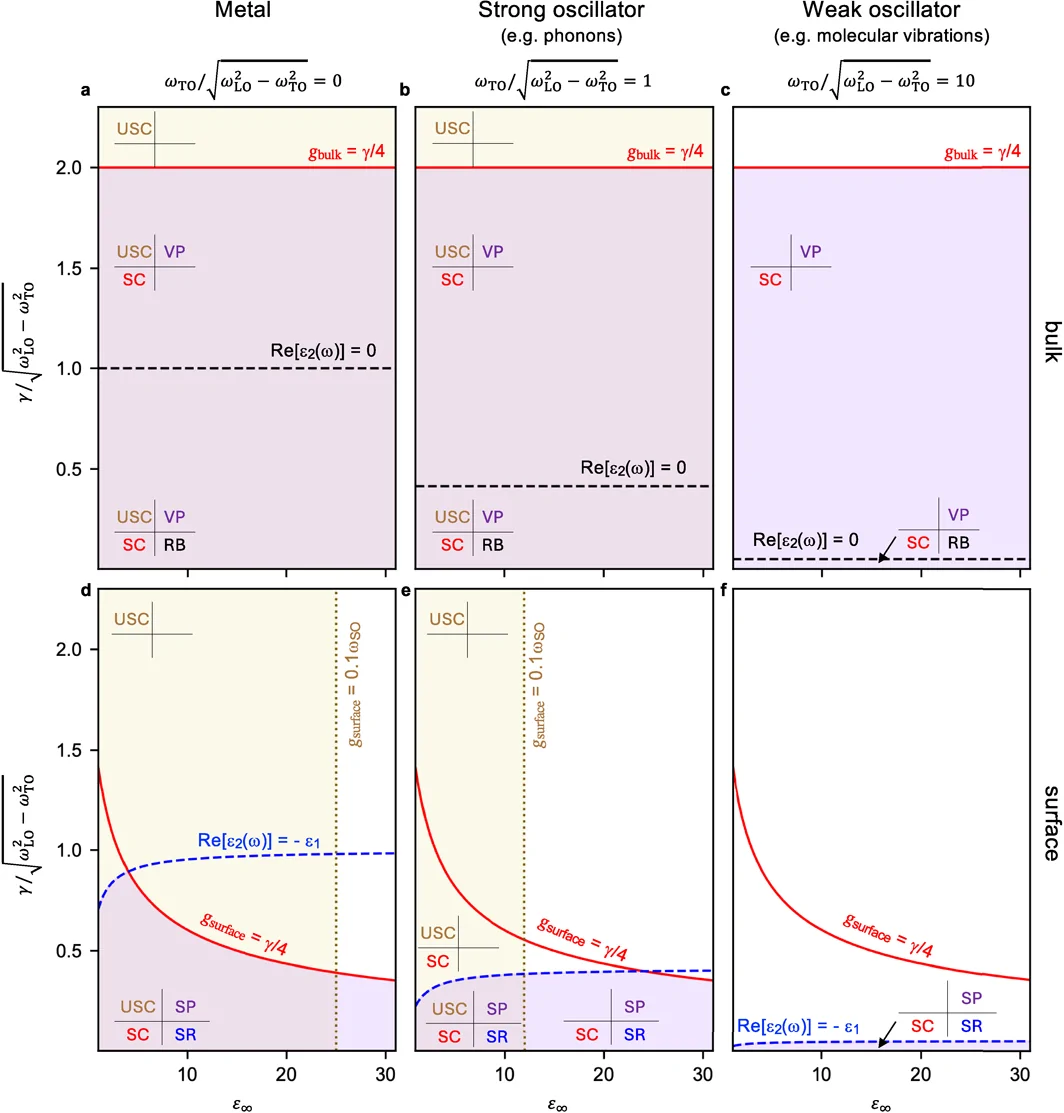
Phase diagrams of light–matter coupling regimes for bulk (a–c) and surface (d–f) polaritons. Columns correspond to three oscillator regimes: (a, d) metal ($\omega_{\text{TO}}=0$, left); (b, e) strong oscillator such as a phonon ($\omega_{\text{TO}}/\sqrt{\left(\omega_{\text{LO}}^{2}-\omega_{\text{TO}}^{2}\right)}=1$, center); and (c, f) weak oscillator such as a molecular vibration ($\omega_{\text{TO}}/\sqrt{\left(\omega_{\text{LO}}^{2}-\omega_{\text{TO}}^{2}\right)}=10$, right). The axes are the normalized damping $\gamma /\sqrt{\left(\omega_{\text{LO}}^{2}-\omega_{\text{TO}}^{2}\right)}$ and the high‐frequency permittivity $\varepsilon_{\infty }$. Solid red lines mark the weak‐to‐strong coupling transition ($g_{\text{bulk}/\text{surface}}=\gamma /4$). Dotted lines indicate the onset of the ultrastrong coupling regime, which is shown by semi‐transparent yellow areas, where $g<\;0.1\omega_{\text{TO}}$ (panels a–c) and $g<\;0.1\omega_{\text{SR}}$ (panels d–f). Dashed black lines in panels a–c mark the onset of Reststrahlen band (RB) formation, occurring below these lines. Dashed blue lines in panels d–f mark the onset of surface resonances, existing below these lines. Purple areas indicate the existence of volume polaritons (VP in panels a–c) and surface polaritons (SPP and SPhP in panels d–f). The 2 × 2 tables inside the individual regions indicate the existence of strong coupling (SC), ultrastrong coupling (USC), Reststrahlen band (RB), volume polariton (VP), surface polaritons (SP), or surface resonance (SR). $\varepsilon_{1}$ = 1 for all the panels.

The red lines indicate the transition from weak to strong coupling, defined by $g_{\text{bulk}}=\gamma /4$ for bulk polaritons and $g_{\text{surface}}=\gamma /4$ for surface polaritons. Because the bulk coupling strength, $g_{\text{bulk}}=\frac{1}{2}\sqrt{\omega_{\text{LO}}^{2}-\omega_{\text{TO}}^{2}},$ is independent of $\varepsilon_{\infty }$, the condition $g_{\text{bulk}}=\gamma /4$ appears as a horizontal line in Figure 3a–c. In contrast, the surface coupling strength depends on $\varepsilon_{\infty }$ through Equation (24), resulting in the curved red lines in Figure 3d–f. The black dashed lines in Figure 3a–c mark the onset of Reststrahlen‐band formation. Below these lines, there exists at least one frequency for which $\text{Re}\left[\varepsilon_{2}\left(\omega \right)\right]=0$. Similarly, the blue dashed lines in Figure 3d–f mark the onset of a surface resonance. Below these lines, there exists at least one frequency for which $\text{Re}\left[\varepsilon_{2}\left(\omega \right)\right]=-\varepsilon_{1}$. Note that the blue lines are obtained by including damping in the condition $\text{Re}\left[\varepsilon_{2}\left(\omega \right)\right]=-\varepsilon_{1}$. Consequently, they are generally not horizontal, unlike the surface‐resonance frequencies defined by Equations (4) and (15), which correspond to the conventional definition of the surface resonance in the absence of damping. The dotted lines indicate the onset of the USC regime. Although the coupling strength is not used as an axis in Figure 3, the USC criterion depends on both the coupling strength and the relevant bare‐mode frequency. Since both quantities depend on $\varepsilon_{\infty }$, varying $\varepsilon_{\infty }$ can move the system into or out of the USC regime.

Figure 3 allows us to assess how SC, USC, RB formation, SRs, and polariton formation are related and whether they necessarily occur together. BrPs form when SC condition is satisfied, whereas confined SPs (SPhP and SPP) can only form when both the surface‐resonance condition (Reε2(ω)=−ε1) and strong coupling (SC) are satisfied. Interestingly, we observe that surface SC and the surface‐resonance condition do not generally coincide and either can be fulfilled without the other being satisfied. For strong oscillators in Figure 3e with $\varepsilon_{\infty }\;≲\;25$, the surface‐resonance condition is the more restrictive requirement and imposes a lower damping threshold than SC alone. For plasmonic matter (Figure 3d), the balance between both conditions depends on $\varepsilon_{\infty }$. For $\varepsilon_{\infty }\;≲\;5$, the surface‐resonance condition remains more restrictive, whereas for $\varepsilon_{\infty }\;≳\;5$, SC becomes the limiting requirement. For weak oscillators (Figure 3f), satisfying the surface‐resonance condition requires significantly lower damping than SC, which explains the limited occurrence of SPs for molecular matter.

Notably, the surface‐resonance condition depends only weakly on $\varepsilon_{\infty }$, whereas surface SC condition becomes increasingly restrictive as $\varepsilon_{\infty }$ increases, which can be attributed to enhanced screening of electric fields below the surface. Comparison with the bulk diagrams (Figure 3a–d) further shows that achieving simultaneously surface SC and a surface resonance requires bulk SC and as consequence the existence of bulk polaritons and the formation of a Reststrahlen band.

Figure 3 further shows that the regions where SPs, VPs, or RBs exist do not necessarily coincide with the USC regime and either can occur without the other. Therefore, USC is neither a necessary nor a sufficient condition for the existence of either surface polaritons, bulk polaritons, or a Reststrahlen band, since it is defined independently of the SC and RB conditions. Nevertheless, for many plasmonic materials and polar crystals, these regimes coincide.

## 4 Conclusion

In this work, we have shown that surface phonon polaritons and surface plasmon polaritons, canonical examples of strong light–matter coupling, can be described within a coupled harmonic oscillator framework based on momentum coupling. By starting from the dispersion relations derived from Maxwell's equations, we demonstrated that these surface polaritons can be described as the result of the strong coupling between a material excitation (a surface phonon or surface plasmon) and a photonic mode identified as the dielectric Brewster mode. Within the MoC model, the conventional strongly confined surface polariton corresponds partially (above TO frequency in case of phonons) or entirely (in case of plasmons) to the lower polariton branch, whereas the complementary upper branch, here termed Brewster polariton, represents a weakly confined surface mode that propagates along the interface.

Our results confirm the MoC model as a powerful tool for analyzing surface polariton dispersions. Beyond reproducing known electromagnetic solutions, the model provides access to key quantities such as the coupling strength and the nature of the bare modes. This framework offers new insight into the surface polaritons and opens a unified route to describing light–matter coupling in strongly confined surface‐wave systems across a wide range of material platforms.

Although the present work focuses on isotropic materials, the approach could in principle be extended to anisotropic systems, where the dielectric response depends on the crystallographic direction. However, in such cases, multiple matter excitations coexist, for example due to distinct resonances along different crystal axes. Since the two‐coupled‐harmonic‐oscillator model employed here only captures the interaction between light and a single matter oscillator, these systems would require a generalized framework involving three or more coupled oscillators, potentially enabling the description of a wider class of hybrid polaritonic phenomena in complex materials [26, 41, 42, 43, 44]. Another interesting direction for future research will be to establish more direct connections between effective coupled‐oscillator descriptions of surface polaritons and recently developed microscopic quantum theories of plasmonic systems, which provide a complementary perspective on ultrastrong light–matter interactions at metal–dielectric interfaces [45].

## 5 Methods

Calculation of the surface polariton field profiles and time averaged Poynting vector:

To calculate the spatial electric field distribution of the surface polaritons in Figure 1d–g and 2d–g, we use the solution of Maxwell's equations for a transverse magnetic wave propagating along the surface. The TM waves can be written as $H=\left(0,H_{y},0\right)$ and $=\left(E_{x},0,E_{z}\right),$ characterized by

(27) $$H_{y,j}\left(x,z\right)=H_{0}e^{ik_{x}x}e^{ik_{z,j}z},$$

(28) $$E_{x,j}\left(x,z\right)=H_{0}\frac{k_{z,j}}{\omega \varepsilon_{0}\varepsilon_{j}}e^{ik_{x}x}e^{ik_{z,j}z},$$

(29) $$E_{z,j}\left(x,z\right)=-H_{0}\frac{k_{x}}{\omega \varepsilon_{0}\varepsilon_{j}}e^{ik_{x}x}e^{ik_{z,j}z},$$

with $k_{x}\left(\omega \right)$ calculated as solution of Equation (1) for real frequency and ω and $k_{z,j}\left(\omega \right)$ as solution of Equation (2).

As a consequence of Equations ((27), (28), (29)), the time averaged Poynting vector is expressed as

(30) $$\langle S_{x,j}\rangle =-\frac{1}{2}\text{Re}\left(E_{z,j}H_{y,j}^{*}\right)=\frac{|H_{0}{|}^{2}}{2\omega \varepsilon_{0}}\text{Re}\left[\frac{k_{x}}{\varepsilon_{j}}\right]e^{-2\text{Im}\left[k_{z,j}\right]},$$

(31) $$\langle S_{y,j}\rangle =0,$$

(32) $$\langle S_{z,j}\rangle =\frac{1}{2}\text{Re}\left(E_{x,j}H_{y,j}^{*}\right)=\frac{|H_{0}{|}^{2}}{2\omega \varepsilon_{0}}\text{Re}\left[\frac{k_{z,j}}{\varepsilon_{j}}\right]e^{-2\text{Im}\left[k_{z,j}\right]}.$$

To facilitate visualization and comparison, the surface polariton field intensities, $|E_{x}\left(x,z\right){|}^{2}$, and the Poynting vector magnitudes, $\left|{\langle S_{x,j}\rangle }^{2}+{\langle S_{x,j}\rangle }^{2}\right|$, are normalized to the maximum value of 1 in each panel.

In Figure 1, Equations ((27), (28), (29), (30), (31), (32)) are solved with $\varepsilon_{1}$ = 1 and $\varepsilon_{2}$ obtained by Equation (3) with parameters $\varepsilon_{\infty }$ = 5, $\nu_{\text{TO}}$ = 800 cm^−1^, $\nu_{\text{LO}}$ = 1000 cm^−1^, and $\gamma_{\text{TO}}$ = 10 cm^−1^.

In Figure 2, Equations ((27), (28), (29), (30), (31), (32)) are solved with $\varepsilon_{1}$ = 1 and $\varepsilon_{2}$ obtained by Equation (13) with parameters $\varepsilon_{\infty }$ = 5, $\nu_{P}$ = 1000 cm^−1^, and $\gamma_{P}$ = 20 cm^−1^.

## Author Contributions

**Edoardo Vicentini:** conceptualization, investigation, writing – review and editing, writing – original draft, funding acquisition. **Nicolas Pajusco:** writing – review and editing, investigation. **Carlos Maciel‐Escudero:** investigation, writing – review and editing. **Xabier Arrieta:** investigation, writing – review and editing. **Javier Aizpurua:** funding acquisition, writing – review and editing. **Ruben Esteban:** investigation, writing – review and editing, funding acquisition. **Rainer Hillenbrand:** conceptualization, investigation, funding acquisition, writing – original draft, writing – review and editing.

## Conflicts of Interest

The authors declare no conflicts of interest.

## Supporting information


Supporting Information S1

## Data Availability

The plotting scripts and complementary description of the calculation used in this study are openly available in the Zenodo repository: https://doi.org/10.5281/zenodo.18714539.
