# Supplementary material for: Description of Surface Polaritons Within a Classical Coupled Harmonic Oscillator Model
Source: Nanophotonics. 2026 Aug 21;15(16):e70238. doi: 10.1002/nap2.70238 (PMC13672150; doi:10.1002/nap2.70238)
Supplement: Supplementary file 1 [file NAP2-15-e70238-s001.docx]

Supporting Information

Description of surface polaritons within a classical coupled harmonic oscillator model

Edoardo Vicentini^*^, Nicolas Pajusco, Carlos Maciel-Escudero, Xabier Arrieta, Javier Aizpurua, Ruben Esteban and Rainer Hillenbrand*.

**Table of Contents**

**Supplementary Notes**

Supplementary Note 1: Derivation of dispersion relations of surface polaritons.

Supplementary Note 2. Discussion over the signs of real and imaginary parts of $k_{z,1}$ and $k_{z,2}$.

Supplementary Note 3. Electrostatic derivation of the surface optical phonon/plasmon eigenfrequency at a planar interface.

Supplementary Note 4. Analytical expression for calculating the phase diagram.

**Supplementary Figures**

Supplementary Figure 1: Graphical representation of the relationship between the arguments of the permittivities.

**Supplementary Tables**

Supplementary Table 1: Summary of bounds for different $\arg[x]$.

### **Supplementary Note 1: Derivation of dispersion relations of surface polaritons.**

In Supplementary Note 1, we summarize the Equations that defines the surface modes at the interface between two distinct media.

We consider a planar interface at $z=0$ between two homogeneous isotropic, non-magnetic media: medium 1 ($z>0$) with permittivity $\varepsilon_{1}(\omega)$ and medium 2 ($z<0$) with permittivity $\varepsilon_{2}(\omega)$.

From Maxwell’s Equations, it can be shown^1^ that electric field $\boldsymbol{E}$ and magnetic field $\boldsymbol{H}$ satisfy the following Helmholtz Equations:

| $\nabla^{2}\boldsymbol{E}\left( r;\omega\right)+ k_{j}^{2} \boldsymbol{E}\left( r;\omega\right)= 0,$  $\nabla^{2}\boldsymbol{H}\left( r;\omega\right)+ k_{j}^{2} \boldsymbol{H}(r;\omega) = 0,$ | (S1a)  (S1b) |
| --- | --- |

with $k_{j}^{2}=\varepsilon_{j}(\omega)k_{0}^{2}$, $k_{0}^{2}=\omega^{2}/c_{0}^{2}$ and and $c_{0}$ the speed of light in vacuum.

The modes of the system can be written as TM waves as $\boldsymbol{H}=(0,\text{ }H_{y},\text{ }0)$ and $\boldsymbol{E}=\left( E_{x},0,E_{z} \right),$ characterised by

| $H_{y,j}\left( x,z \right)=H_{0}e^{ik_{x}x}e^{ik_{z,j}z},$  $E_{x,j}\left( x,z \right)=H_{0}\frac{k_{z,j}}{\omega\varepsilon_{0}\varepsilon_{j}}e^{ik_{x}x}e^{ik_{z,j}z},$  $E_{z,j}\left( x,z \right)=-H_{0}\frac{k_{x}}{\omega{\varepsilon_{0}\varepsilon}_{j}}e^{ik_{x}x}e^{ik_{z,j}z},$ | (S2a)  (S2b)  (S2c) |
| --- | --- |

with

$k_{z,j}^{2}=\varepsilon_{j}k_{0}^{2}-k_{x}^{2}.$

(S3)

We note that in many textbooks the signs of the exponential terms $e^{ik_{z,j}z}$ in Equation S2 differ between the two media (typically written as $+ik_{z,1}z$ and $-ik_{z,2}z$). We emphasize that our choice, like any alternative, does not impose additional constraints on the solution; it only defines the convention for the propagation direction in each medium. Accordingly, $k_{z,j}$ may still take positive or negative real and imaginary parts, allowing fields to propagate and/or decay in either direction. For example, within the convention adopted in Equation S2, for $z>0$, if $\mathrm{Re}(k_{z,1})>0$ the wave propagates away from the surface toward infinity, whereas if $\mathrm{Re}(k_{z,1})<0$ it propagates from infinity toward the surface. The physically correct signs of $k_{z}$, corresponding to the eigenmodes of the system, are determined by the Sommerfeld radiation condition^1^. This condition imposes fields decaying exponentially away from the interface; namely, $\mathrm{Im}(k_{z,1})>0$ and $\mathrm{Im}(k_{z,2})<0$. In Supplementary Note 2, we show that a mode satisfying these conditions exists for both negative and positive values of $\varepsilon_{2}$; that is, for both SPhPs/SPPs and Brewster polaritons.

Boundary conditions at $z=0$ require continuity of $H_{y}$ and $E_{x}$:

| $H_{y,1}\left( z=0 \right)=H_{y,2}\left( z=0 \right),$ $E_{x,1}\left( z=0 \right)=E_{x,2}\left( z=0 \right),$ | (S4a)  (S4b) |
| --- | --- |

These conditions lead to

$$\frac{k_{z,1}}{\varepsilon_{1}}=\frac{k_{z,2}}{\varepsilon_{2}}.$$

(S5)

Solving for the in-plane and out-of-plane wavevectors yields the surface polariton dispersion relations:

| $k_{x}^{2}=\frac{\varepsilon_{1}\varepsilon_{2}}{\varepsilon_{1}+\varepsilon_{2}}k_{0}^{2},$  $k_{z,1}^{2}=\frac{\varepsilon_{1}^{2}}{\varepsilon_{1}+\varepsilon_{2}}k_{0}^{2},$  $k_{z,2}^{2}=\frac{\varepsilon_{2}^{2}}{\varepsilon_{1}+\varepsilon_{2}}k_{0}^{2}$. | (S6a)  (S6b)  (S6c) |
| --- | --- |

The dispersion relation of Equations (S6) relates the square of $k_{z,1}$ and $k_{z,2}$ and $\varepsilon_{1}$ and $\varepsilon_{2}$, however these expressions alone do not uniquely define the signs of the solutions. In Supplementary Note 2, we will discuss which sign of real and imaginary parts of $k_{z,1}$ and $k_{z,2}$ are possible.

**Supplementary Note 2. Discussion over the signs of real and imaginary parts of** $\boldsymbol{k}_{\boldsymbol{z,1}}$ **and** $\boldsymbol{k}_{\boldsymbol{z,2}}$**.**

In Supplementary Note 2, we discuss which signs of real and imaginary parts of $k_{z,1}$ and $k_{z,2}$ are possible according to boundary conditions and Sommerfield radiation condition. In this way, we show that surface phonon/plasmon polariton and Brewster polariton are valid solutions of Maxwell’s Equation at the boundary between two media.

To be defined as eigenmodes, solution of Maxwell’s Equations must also fulfil the Sommerfield radiation condition, which for a surface mode, it is equivalent to have fields that exponentially decay away from the surface^1^. According to the fields defined in Equation S2 an, Sommerfield radiation condition impose $\mathrm{Im}[k_{z,1}]>0$ and $\mathrm{Im}\left[ k_{z,2} \right]<0$. In Supplementarty Note 1, we retrieved the relation between the square of $k_{z,1}$ and $k_{z,2}$ and $\varepsilon_{1}$ and $\varepsilon_{2}$ (Equation S6b and Equation S6c), however these expressions do not uniquely define the signs of $\mathrm{Im}[k_{z,1}]$ and $\mathrm{Im}[k_{z,1}]$.

In the following, we analyse the sign of $k_{z,1}$ and $k_{z,2}$ and verify that they fulfil Sommerfield radiation condition.

From Equation S6b and Equation S6c, the possible sign of $k_{z,1}$ and $k_{z,2}$ are

| $k_{z,1}=+k_{0}\sqrt{\frac{\varepsilon_{1}^{2}}{\varepsilon_{1}+\varepsilon_{2}}} ,$  $k_{z,1}=-k_{0}\sqrt{\frac{\varepsilon_{1}^{2}}{\varepsilon_{1}+\varepsilon_{2}}} ,$  $k_{z,2}=+k_{0}\sqrt{\frac{\varepsilon_{2}^{2}}{\varepsilon_{1}+\varepsilon_{2}}} ,$  $k_{z,2}=-k_{0}\sqrt{\frac{\varepsilon_{2}^{2}}{\varepsilon_{1}+\varepsilon_{2}}} .$ | (S7a)  (S7b)  (S7c)  (S7d) |
| --- | --- |

Since we are only interested in the signs of the real and imaginary part of $k_{z,j}$, it is simpler to work with the polar form $z=\left| z \right|e^{i\arg\{z\}}$ with $\arg\{z\}$ the argument of the complex number *z*, such that we can analyze $\arg\{k_{z,j}\}$ to determine the signs of real and imaginary parts of $k_{z,j}$. Additionally, to simplify the notation, we define the quantities

| $\alpha_{1}= \arg\{\varepsilon_{1}\},$  $\alpha_{2}= \arg\left\{ \varepsilon_{2} \right\},$  $\theta_{1}= \arg\left\{ k_{z,1} \right\},$  $\theta_{2}= \arg\{k_{z,2}\}.$ | (S8a)  (S8b)  (S8c)  (S8d) |
| --- | --- |

**Supplementary Figure 1:** Graphical representation of the relationship between $\alpha_{2}=\arg\left\{ \varepsilon_{2} \right\}$ (black line), $2\gamma=\arg\left\{ \varepsilon_{1}+\varepsilon_{2} \right\}$ (red line), $\gamma=\arg\left\{ \varepsilon_{1}+\varepsilon_{2} \right\}/2$ (orange line) and $\alpha-\gamma=\arg\left\{ \varepsilon_{2} \right\}-\arg\left\{ \varepsilon_{1}+\varepsilon_{2} \right\}/2$ (blue line) for three different values of $\varepsilon_{1}$ and $\varepsilon_{2}$. **a** ${Re[\varepsilon}_{2}]$ positive. **b** ${Re[\varepsilon}_{2}]$ negative, ${Re[\varepsilon}_{1}]$ positive and small. **c** ${Re[\varepsilon}_{2}]$ negative, ${Re[\varepsilon}_{1}]$ positive and large. **a,b,c** $\varepsilon_{2}$ is indicated as a black dot, $\varepsilon_{1}+\varepsilon_{2}$ is indicated as red dot, while the dashed line indicates the sum operation. ${Im[\varepsilon}_{1}]=0$

With this new notation, the Sommerfield radiation condition, $\mathrm{Im}[k_{z,1}]>0$ and $\mathrm{Im}\left[ k_{z,2} \right]<0$, can be expressed as

| $0<\theta_{1}<\pi,$  $-\pi<\theta_{2}<0.$ | (S9a)  (S9b) |
| --- | --- |

In this note, we analyze the typical surface polariton scenario, where the upper half space is either vacuum or a constant lossless dielectric (which correspond to $\mathrm{Re}\left( \varepsilon_{1} \right)>0$ and $\mathrm{Im}\left( \varepsilon_{1} \right)=0$) and the lower half space a lossy phononic/plasmonic material ($\mathrm{Im}\left( \varepsilon_{2} \right)>0$ and $\mathrm{Re}\left( \varepsilon_{2} \right)$ positive or negative). These conditions imply $\alpha_{1}= 0$ and $0<\alpha_{2}<\pi$. Same results can also be obtained if $\mathrm{Im}\left( \varepsilon_{1} \right)\ll Im\left( \varepsilon_{2} \right)$.

We first analyze $k_{z,1}$. From Equation S7a and S7b, with $\varepsilon_{1}$ and $k_{0}$ are real and positive quantities, we can express $\theta_{1}$, respectively as

| $\theta_{1}=-\frac{\arg\left\{ \varepsilon_{1}+\varepsilon_{2} \right\}}{2}=-\gamma,$  or  $\theta_{1}=-\frac{\arg\left\{ \varepsilon_{1}+\varepsilon_{2} \right\}}{2}+\pi=-\gamma+\pi.$ | (S10a)  (S10b) |
| --- | --- |

where we introduced the quantity $\gamma=\frac{\arg\left\{ \varepsilon_{1}+\varepsilon_{2} \right\}}{2}$. Since adding a positive real number $\varepsilon_{1}$ to a complex number $\varepsilon_{2}$ shifts it along the real axis in the complex plane (see example in Supplementary Figure 1), we can derive that

$$0<\arg\left\{ \varepsilon_{1}+\varepsilon_{2} \right\}<\arg\left\{ \varepsilon_{2} \right\}<\pi,$$

(S11)

which implies

$$0<\gamma<\frac{\pi}{2}.$$

(S12)

Combining S12 with Equation S10, we find that

| ${-\frac{\pi}{2}<\theta}_{1}<0,$  or  ${\frac{\pi}{2}<\theta}_{1}<\pi.$ | (S13a)  (S13b) |
| --- | --- |

Since Sommerfield radiation condition imposes $0<\theta_{1}<\pi$, we retrieved that only Equation (S10b) and as consequence Equation (S7b) provide the correct sign of $k_{z,1}$. $k_{z,1}$ is thus characterized by a negative real part and a positive imaginary part.

Having identified the physically allowed $k_{z,1}$, we determine the solution of $k_{z,2}$ which satisfy the boundary conditions (S5) and we later verify that it also fulfills Sommerfield radiation condition.

We rewrite Equation S5 in the form

$$k_{z,2}=\frac{\varepsilon_{2}}{\varepsilon_{1}}k_{z,1} ,$$

(S14)

and we analyze $\theta_{2}=\arg\left\{ k_{z,2} \right\}$. Since $\varepsilon_{1}$ is real and positive, we can rewrite

$$\theta_{2}=\alpha_{2} +\theta_{1},$$

(S15)

and substituting the solution for $\theta_{1}$ from Equation (S10b), we obtain

$$\theta_{2}=\alpha_{2}-\gamma+\pi.$$

(S16)

Equation (S16) provides the solution of $k_{z,2}$ compatible with boundary conditions. Now we verify that Sommerfield radiation condition (Equation (S9)) is also fulfilled.

We know that $0<\alpha_{2}<\pi$ and from the Equation (S12) that $0<\gamma<\frac{\pi}{2}$, together these relations imply

$$0<\alpha_{2}-\gamma<\alpha_{2}<\pi,$$

(S17)

which set the conditions for $\theta_{2}$ as

$$\pi<\theta_{2}<2\pi,$$

(S18)

or equivalently (modulus $2\pi$)

$$-\pi<\theta_{2}<0.$$

(S19)

which is exactly the Sommerfield radiation condition for $k_{z,2}$.

As a final note, we want to highlight that in case of $\mathrm{Im}\left( \varepsilon_{1} \right)=0$, $\mathrm{Im}\left( \varepsilon_{2} \right)=0$ and $\mathrm{Re}\left( \varepsilon_{2} \right)>0$, no solution fulfill the Sommerfield radiation condition.

In summary, we demonstrated that in case of $\varepsilon_{1}$ real and positive and $\varepsilon_{2}$ characterized by a strictly positive imaginary part, it is always possible to find Maxwell’s Equation solutions that fulfill Sommerfield radiation condition. As consequence, SPhP, SPP and Brewster polariton are properly defined modes, on the other hand the dielectric Brewster mode is properly defined only in the presence of a lossy medium.

| Argument | Angles | Lower Bound | Upper bound |
| --- | --- | --- | --- |
| $\arg\left\{ k_{z,1} \right\}$ | $\theta_{1}$ | 0 | $\pi$ |
| $\arg\left\{ k_{z,2} \right\}$ | $\theta_{2}$ | $-\pi$ | 0 |
| $\arg\left\{ \varepsilon_{1} \right\}$ | $\alpha_{1}$ | $0$ | $0$ |
| $\arg\left\{ \varepsilon_{2} \right\}$ | $\alpha_{2}$ | $0$ | $\pi$ |
| $\arg\left\{ \varepsilon_{2}+\varepsilon_{1} \right\}$ | $2\gamma$ | $0$ | $\pi$ |
| $\frac{\arg\left\{ \varepsilon_{2}+\varepsilon_{1} \right\}}{2}$ | $\gamma$ | $0$ | $\frac{\pi}{2}$ |
| $-\frac{\arg\left\{ \varepsilon_{2}+\varepsilon_{1} \right\}}{2}$ | $-\gamma$ | $-\frac{\pi}{2}$ | 0 |
| $-\frac{\arg\left\{ \varepsilon_{2}+\varepsilon_{1} \right\}}{2}+\pi$ | $\pi-\gamma$ | $\frac{\pi}{2}$ | $\pi$ |
| $\arg\left\{ \varepsilon_{2} \right\}-\frac{\arg\left\{ \varepsilon_{2}+\varepsilon_{1} \right\}}{2}$ | $\alpha_{2}-\gamma$ | $0$ | $\pi$ |
| $\arg\left\{ \varepsilon_{2} \right\}-\frac{\arg\left\{ \varepsilon_{2}+\varepsilon_{1} \right\}}{2}+\pi$ | $\alpha_{2}-\gamma+\pi$ | $-\pi$ | 0 |

**Supplementary Table 1:** Summary of bounds for different $\arg[x]$.

**Supplementary Note 3. Electrostatic derivation of the surface optical phonon/plasmon eigenfrequency at a planar interface**

In Supplementary Note 3, we derive the surface phonon/plasmon resonance as solution of the electrostatic Maxwell’s equations.

We consider a planar interface at $z=0$ between two media: medium 1 ($z>0$) with permittivity $\varepsilon_{1}$ and medium 2 ($z<0$) with frequency-dependent permittivity $\varepsilon_{2}(\omega)$. In the electrostatic approximation ($\nabla\times\boldsymbol{E}\left( \boldsymbol{r},\omega\right)=0$), the electric field is curl-free and can be expressed as the gradient of a scalar potential:

$$\boldsymbol{E}\left( \boldsymbol{r},\omega\right)=-\nabla\phi\left( \mathbf{r},\omega\right).$$

(S20)

In charge-free, homogeneous regions ($\rho_{f}=0$), Maxwell’s equation reduces to Laplace’s equation for the potential, that is,

$$\nabla^{2}\phi\left( \mathbf{r},\omega\right)=0.$$

(S21)

Surface-bound modes are localized near the interface and decay exponentially into both media. Accordingly, one expects solutions of Eq. (S21) to take the following form

| $\phi_{1}\left( x,y,z,\omega\right)=\Phi_{1}\left( x,y,\omega\right)e^{-\kappa z},z>0,$ $\phi_{2}(x,y,z,\omega)=\Phi_{2}(x,y,\omega)e^{\kappa z},z<0,$ | (S22a)  (S22b) |
| --- | --- |

where $\kappa>0$ to ensure spatial localization of the field. The normal components of the electric field at the interface ($z=0)$ follow directly from the potentials, as

| $E_{z,1}(z=0)=-\partial_{z}\phi_{1}(z=0)=\kappa\Phi_{1},$ $E_{z,2}(z=0)=-\partial_{z}\phi_{2}(z=0)=-\kappa\Phi_{2}.$ | (S23a)  (S23b) |
| --- | --- |

The boundary conditions at the interface require continuity of the potential ($\phi_{1}=\phi_{2}$) and continuity of the normal component of the displacement field ($\hat{z}\cdot(\boldsymbol{D}_{1}-\boldsymbol{D}_{2})=0$), giving

| $\Phi_{1}=\Phi_{2}\equiv\Phi,$ $\varepsilon_{1}E_{z,1}-\varepsilon_{2}\left( \omega\right)E_{z,2}=0.$ | (S24a)  (S24b) |
| --- | --- |

These two relations, together with Eqs. (S23a)-(S23b), leads to the **surface mode condition:**

$$\varepsilon_{2}(\omega_{s})+\varepsilon_{1}=0.$$

(S25)

For a polar dielectric with a single infrared-active phonon, described by $\varepsilon_{2}(\omega)=\varepsilon_{\infty}\frac{\omega^{2}-\omega_{\mathrm{LO}}^{2}}{\omega^{2}-\omega_{\mathrm{TO}}^{2}}$, the surface phonon resonance frequency is

$$\omega_{\mathrm{SR}}^{2}=\frac{\varepsilon_{\infty}\omega_{\mathrm{LO}}^{2}+\varepsilon_{1}\omega_{\mathrm{TO}}^{2}}{\varepsilon_{1}+\varepsilon_{\infty}}.$$

(S26)

For a metal with $\varepsilon_{2}(\omega)=\varepsilon_{\infty}\left( 1-\frac{\omega_{P}^{2}}{\omega^{2}} \right)$, the surface plasmon resonance frequency is

$$\omega_{\mathrm{SR}}^{2}=\frac{\varepsilon_{\infty}}{\varepsilon_{\infty}+\varepsilon_{1}}\text{ }\omega_{P}^{2}.$$

(S27)

**Supplementary Note 4. Analytical expression for calculating the phase diagram**

We derive the analytical expression for the different regions of the phase diagrams of light–matter coupling present in Figure 3 of the main text.

The SC and USC conditions are defined by $\gamma/4<g$ and $0.1\text{ }\omega_{2}<g$, respectively.

For bulk, $g_{\mathrm{bulk}}=\frac{1}{2}\sqrt{\omega_{\mathrm{LO}}^{2}-\omega_{\mathrm{TO}}^{2}}$ and $\omega_{2}=\omega_{\mathrm{TO}}$, in this way the bulk SC condition becomes

$$\frac{\gamma}{\sqrt{\omega_{\mathrm{LO}}^{2}-\omega_{\mathrm{TO}}^{2}}}<2,$$

(S28)

and the bulk USC condition becomes

$$\frac{\omega_{\mathrm{TO}}}{\sqrt{\omega_{\mathrm{LO}}^{2}-\omega_{\mathrm{TO}}^{2}}}<5.$$

(S29)

For the surface polaritons, $g_{\mathrm{surface}}=g_{\mathrm{bulk}}\sqrt{\frac{\varepsilon_{1}}{{\varepsilon_{1}+\varepsilon}_{\infty}}}$ and $\omega_{2}=\sqrt{\frac{\varepsilon_{\infty}\text{ }\omega_{\mathrm{LO}}^{2}+\varepsilon_{1}\text{ }\omega_{\mathrm{TO}}^{2}}{\varepsilon_{1}+\varepsilon_{\infty}}}$ . These expressions yield the surface SC condition

$$\frac{\gamma}{\sqrt{\omega_{\mathrm{LO}}^{2}-\omega_{\mathrm{TO}}^{2}}}<2\sqrt{\frac{\varepsilon_{1}}{{\varepsilon_{1}+\varepsilon}_{\infty}}},$$

(S30)

and the surface USC condition

$$\varepsilon_{\infty}<\varepsilon_{1}\frac{\left( 25-\left( \frac{\omega_{\mathrm{TO}}}{\sqrt{\omega_{\mathrm{LO}}^{2}-\omega_{\mathrm{TO}}^{2}}} \right)^{2} \right)}{\left( 1+\left( \frac{\omega_{\mathrm{TO}}}{\sqrt{\omega_{\mathrm{LO}}^{2}-\omega_{\mathrm{TO}}^{2}}} \right)^{2} \right)}.$$

(S31)

The condition that ensures the existence of a surface resonance also in case of damping is obtained from the existence of $\mathrm{Re}\left[ \varepsilon_{2}\left( \omega\right) \right]=-\varepsilon_{1}$. Substituting Eq. 3 gives

$$\varepsilon_{\infty}\left( \frac{\left( \omega_{\mathrm{LO}}^{2}-\omega^{2} \right)\left( \omega_{\mathrm{TO}}^{2}-\omega^{2} \right)+\gamma^{2}\omega^{2}}{\left( \omega_{\mathrm{TO}}^{2}-\omega^{2} \right)^{2}+\gamma^{2}\omega^{2}} \right)=-\varepsilon_{1},$$

(S32)

a quadratic equation in $\omega^{2}$, which admits real solutions if its discriminant is nonnegative, leading to the condition

$$\frac{\gamma}{\sqrt{\omega_{\mathrm{LO}}^{2}-\omega_{\mathrm{TO}}^{2}}}<\sqrt{\left( \frac{\omega_{\mathrm{TO}}}{\sqrt{\omega_{\mathrm{LO}}^{2}-\omega_{\mathrm{TO}}^{2}}} \right)^{2}+\frac{\varepsilon_{\infty}}{\varepsilon_{1}+\varepsilon_{\infty}}}-\frac{\omega_{\mathrm{TO}}}{\sqrt{\omega_{\mathrm{LO}}^{2}-\omega_{\mathrm{TO}}^{2}}}.$$

(S33)

The condition for the existence of a Reststrahlen band, $\left[ \varepsilon_{2}\left( \omega\right) \right]=0$, follows from Equation S33 by setting $\varepsilon_{1}=0$, yielding

$$\frac{\gamma}{\sqrt{\omega_{\mathrm{LO}}^{2}-\omega_{\mathrm{TO}}^{2}}}<\sqrt{\left( \frac{\omega_{\mathrm{TO}}}{\sqrt{\omega_{\mathrm{LO}}^{2}-\omega_{\mathrm{TO}}^{2}}} \right)^{2}+1}-\frac{\omega_{\mathrm{TO}}}{\sqrt{\omega_{\mathrm{LO}}^{2}-\omega_{\mathrm{TO}}^{2}}}.$$

(S34)

**Supplementary References**

1.	Le Ru, E. C. & Etchegoin, P. G. *Principles of Surface-Enhanced Raman Spectroscopy: And Related Plasmonic Effects*. (Elsevier, Amsterdam Heidelberg, 2011).
